# T40. GPR52 AGONISTS REPRESENT A NOVEL APPROACH TO TREAT UNMET MEDICAL NEED IN SCHIZOPHRENIA

**DOI:** 10.1093/schbul/sby016.316

**Published:** 2018-04-01

**Authors:** Andrew J Grottick, Ben Grayson, Giovanni Podda, Nagi Idris, Cornelia Dorner-Ciossek, Joanna Neill, Scott Hobson

**Affiliations:** 1 Beacon Discovery, Inc; 2 University of Manchester; 3 Boehringer Ingelheim Pharma

## Abstract

**Background:**

There are currently no treatment options for key symptom domains in certain psychiatric and neurological diseases. For example, antipsychotics effectively treat the positive symptoms of schizophrenia, however both the cognitive impairments associated with schizophrenia (CIAS) and negative symptoms, both key predictors of functional outcome, are not treated by current therapies. Additionally, psychotic symptoms associated with neurological diseases such as Alzheimer’s Disease (AD) are not adequately treated with current antipsychotics. Therefore, novel mechanisms to address these unmet medical needs are urgently required and are under investigation.

GPR52 is a Gs-coupled orphan g-protein coupled receptor which has an intriguing pattern of brain expression. In cortex, GPR52 is expressed primarily on glutamatergic neurons and co-localized with the Gs-coupled D1 receptor (D1R). Deficiencies in D1R activation are associated with both cognitive deficit and negative symptoms of schizophrenia. In contrast, in the striatum, GPR52 is almost exclusively co-expressed with the Gi-coupled D2 receptor (D2R), which mediates the reduction in positive symptoms by antipsychotics. Based on GPR52’s functional coupling and co-localization, agonists may be predicted to resemble D1R agonists in cortical regions, thus treating cognitive or negative symptoms, while resembling D2R antagonists in striatal regions. Thus, GPR52 agonists have the potential to provide a novel therapeutic strategy for the currently untreated CIAS and negative symptom domains in addition to the psychotic symptoms of AD.

**Methods:**

To assess the antipsychotic potential of GPR52 agonists, they were tested for their ability to decrease psychostimulant-induced hyperlocomotion. The efficacy of GPR52 agonists for CIAS and sociability, an aspect of negative symptoms, was assessed in the sub-chronic phencyclidine (scPCP) model for schizophrenia, known to induce long-lasting cognitive and social behaviour deficits, in addition to a reduction in parvalbumin-positive GABAergic interneurons in hippocampus and pre-frontal cortex. Rats were treated with PCP twice daily for 7 days followed by 7 days washout and then tested in the attentional set shifting task (ASST) for executive function and the social interaction test for sociability respectively following treatment with a GPR52 agonist.

**Results:**

GPR52 agonist 1 dose-dependently reversed psychostimulant-induced hyperlocomotion in rats at doses which were behaviorally quiescent when administered alone. Additionally, GPR52 agonist 2 showed a robust, dose-dependent rescue of scPCP induced deficits in the extra dimensional shift phase of the ASST, achieving significance after a 4 mg/kg p.o. application. Likewise, GPR52 agonist 2 significantly rescued scPCP induced deficits in social interaction at identical doses as in ASST without effects on object exploration or locomotor activity.

**Discussion:**

GPR52 agonists were efficacious in animal models assessing the three main symptoms domains associated with schizophrenia. Efficacy in ASST and SI demonstrate both pro-cognitive efficacy and restoration of an aspect of negative symptoms, respectively, in a well-established model inducing behavioral and neuropathological deficits associated with schizophrenia. Furthermore, GPR52 agonists reduced psychostimulant-induced hyperlocomotion, an effect associated with antipsychotic efficacy. Taken together, these data demonstrate the potential of this innovative mechanism to simultaneously treat the three core symptoms domains of schizophrenia as well as potentially treat the psychotic symptoms associated with other neurological disorders.

